# Comparison of amount of postoperative use of blood and blood products between patients receiving and not receiving salicylate before coronary surgery

**DOI:** 10.1186/1749-8090-8-S1-P80

**Published:** 2013-09-11

**Authors:** B Ozcem, U Yetkin, M Bademci, M Akyuz, S Yazman, E Celik, N Karakas, I Yurekli, A Gurbuz

**Affiliations:** 1 Department of Cardiovascular Surgery, Izmir Katip Celebi University Ataturk Training and Research Hospital, Izmir, Turkey

## Background

In this study, we aimed to present whether there was a significant difference between patients receiving 100 mg salicylate and not receiving salicylate before coronary artery bypass grafting in terms of amount of postoperative use of blood and blood products.

## Methods

Sixty-one patients that underwent coronary bypass surgery in one year at our clinic were investigated retrospectively. Thirty (49.2%) of them were receivers of 100 mg enteric-coated salicylate and 31 (50.8%) were non-receivers, divided into 2 groups. The mean age of salicylate receivers was 61.33 years whereas it was 57.71 years in the non-receivers.

## Results

There was no significant difference between groups that underwent on-pump coronary bypass surgery in terms of mean units of blood and blood products used (p>0.05). The percentage of blood product use was 54.5% in salicylate receivers and 55.6% in non-receivers. There was no significant difference between groups of patients that underwent off-pump beating heart coronary surgery in terms of amount of blood and blood product used (p>0.05). The percentage of blood product use was 37.5% in salicylate receivers and 50% in non-receivers in this group of patients (p>0.05).

## Conclusions

When the amount blood and blood products used was investigated for patients undergoing either on-pump or off-pump coronary bypass in terms of salicylate use, no significant difference was detected between groups.

